# Health-Related Quality of Life after Pediatric Liver Transplantation: A Qualitative Analysis of the Perspectives of Health Care Providers

**DOI:** 10.1155/2017/5274923

**Published:** 2017-06-20

**Authors:** Mar Miserachs, David B. Nicholas, Anthony R. Otley, Vicky Lee Ng

**Affiliations:** ^1^Transplant and Regenerative Medicine Centre, Division of Pediatric Gastroenterology, Hepatology and Nutrition, The Hospital for Sick Children, University of Toronto, Toronto, ON, Canada; ^2^Faculty of Social Work, University of Calgary, Calgary, AB, Canada; ^3^Division of Gastroenterology and Nutrition, Department of Pediatrics, IWK Health Centre, Halifax, NS, Canada; ^4^Department of Pediatrics, Faculty of Medicine, Dalhousie University, Halifax, NS, Canada

## Abstract

With improved survival outcomes after pediatric liver transplantation (LT), health-related quality of life (HRQoL) is an important outcome metric. Understanding the elements contributing to HRQoL after LT in children would enable more targeted strategies towards optimizing best outcomes. This qualitative study aimed to explore health care providers (HCP) perceptions about HRQoL after pediatric LT. Thirteen experienced HCP participated in two focus group discussions. Data analysis via a thematic analysis approach revealed 4 major themes: “LT as a facilitator of better HRQoL,” “coping and adapting to LT,” “living with a transplanted liver,” and “the family context.” HCP identified elements that both enhance (improved physical health, peer relationship, and activities of daily living) and challenge (need for immunosuppression, transplant follow-up, and restrictions) the multidimensional domains of HRQoL. HCP perceived LT to be a stressful life-changing event for children and their families. Patients and their parents' ability to cope and adjust positively to LT was perceived as a key contributor to better HRQoL. HCP perspective highlights the importance of promoting psychosocial support and a family-centered care delivery model towards the overarching goal of optimizing durable outcomes.

## 1. Introduction

Advances in surgical, anesthesia, and intensive-care techniques, as well as early and long-term medical aftercare, have led to current excellent patient survival rates following pediatric liver transplantation (LT) [1]. With long-term survival now the rule rather the exception, patient-reported outcomes such as the construct of Health-Related Quality of Life (HRQoL) have become the focus of quantitative clinical research [2]. Studies on HRQoL in pediatric LT recipients most frequently assess self- and parent-reported HRQoL determinations via the utilization of validated age-appropriate instruments and have explored the associations between demographic or medical variables and HRQoL outcomes [3, 4]. Two systematic reviews evaluating HRQoL outcomes in pediatric LT recipients underscore HRQoL being lower than healthy controls, although comparable to children with chronic diseases or other pediatric solid organ transplant recipients [5, 6]. The more recent systematic review by Parmar et al. [5] highlighted the contribution to the field of newer disease-specific tools, although interventional studies targeting strategies to address this decreased HRQoL remain sparse [5]. Additional ways to better understand the challenges experienced by this patient population are needed to enhance our ability to derive novel strategies.

Qualitative research has established itself in the field of solid organ transplantation by providing insights into our current understanding about health, the illness experience, and the effectiveness of health care [7]. Qualitative research studies addressing HRQoL after pediatric LT have explored the views and experiences of children, adolescents, and young adults following LT and during transition [8–12]. Analysis of the perspectives of health care providers (HCP) on HRQoL following pediatric LT has not been reported. Available data from a qualitative study involving HCP in two quaternary academic health sciences centers with dedicated time and expertise in providing care to pediatric LT patients were analyzed. Herein, the objectives of this study were to describe HCP perceptions about HRQoL of pediatric LT recipients.

## 2. Material and Methods

Following institutional ethics board approval, semistructured focus group interviews with HCP representing the wide spectrum of interdisciplinary team members directly involved in the clinical care of the pediatric LT recipient were completed. An interview guide with open-ended questions was developed based on an extensive literature review (Box 1). Purposive sampling was used to recruit 6 to 10 participants in each focus group to allow for well-rounded discussions with ample time for each participant to convey their individual perspectives yet sufficient opportunity for discussion [13–15]. Interdisciplinary HCP with a minimum of 3 years of experience in the care of pediatric LT patients at two Canadian institutions were invited to participate in focus groups. Within each focus group, a trained facilitator led the discussion, while an observer monitored, noted group process, and attended to any issues and questions as they emerged. Interviews were audio recorded and subsequently transcribed verbatim in preparation for analysis.

### 2.1. Data Analysis

Focus group transcripts were subjected to established methods of qualitative thematic analysis approach, guided by McCracken's long interview method [16], that comprised a multistep process of (1) line-by-line review and code identification for salient constructs within individual transcripts, (2) identification of convergent and divergent codes across transcripts, (3) integration of codes across participant cohorts, and (4) solidification of themes following the extensive review of the above three steps, demonstrating saturation of themes [16].

Data were coded by trained qualitative research analysts. Further, members of the research team independently reviewed and coded a portion of the data to ensure interrater reliability and consistency among the primary coder. The remainder of the coding was completed by the trained research coders, under the supervision of an experienced qualitative research analyst and methodologist. A minimum of two team members analyzed all transcripts independently, and any discrepancies in coding were resolved through consensus. A database management and computer software system for qualitative data analysis was utilized (NVivo™ 2.0 QSR International Pty. Ltd., 2002).

Data were coded for categories and themes that depicted components of HRQoL for children with a liver transplant. Guiding this process, the team met regularly to review the analysis process. Accordingly, a systematic, rigorous process of qualitative analysis was performed. Methodological rigor included means of trustworthiness, namely, interrater reliability as noted above, peer debriefing which consisted of a review, and confirmation of findings by experienced clinicians and other experts (e.g., researchers) in this field. Thick description of findings was demonstrated and is reflected with reference to text quotes in the depiction of study findings.

## 3. Results

A total of 13 (100% female) HCP participated in one of two focus group discussions, each at least one hour in duration. The focus groups comprised 6 and 7 HCP, respectively. HCP participant demographic data are provided in Table 1. To ensure participant confidentiality, sociodemographic or professional information was not linked to corresponding text quotes from the data.

Concepts emerging from analysis spanned four domains: (1) LT as a facilitator of better HRQoL; (2) coping and adapting to LT; (3) living with a transplanted liver; and (4) the family context. Corroborating text quotes are provided within the text.

### 3.1. LT as a Facilitator of Better HRQoL

#### 3.1.1. Enhanced Physical Health

HCP perceived enhanced physical health following LT, to be a key facilitator of HRQoL, especially in patients with chronic cholestatic liver disease or decompensated cirrhosis. Resolution of debilitating effects, such as malnutrition, fatigue, and/or impaired growth and neurodevelopment, and improvement in the visible signs of liver disease such as jaundice or ascites were linked to a positive impact on patient's body image and self-esteem.

A lot of kids return to school with changed body image including resolved ascites and changed skin color (resolved jaundice).

(After liver transplant) kids come back to clinic with improved energy level, appetite and sleeping patterns. They are able to keep up with their friends.

After transplant, when their nutritional status is better, they seem much more interactive than they were pre-transplant, much happier to play….

The thrill you can see in parents when after transplant, their children, start to meet the milestones that they were lagging behind.

#### 3.1.2. Activities of Daily Living and Peer Relationships

A restored ability to participate in activities of daily living, such as attending school or practicing sports with age-matched peers, was highlighted. Both peer-peer relationships and being perceived and treated as equal to peers and siblings were thought to nurture a positive effect on HRQoL.

Probably one of the best things for them is to be back at school, to be playing hockey again, which is all tied up with the fact that they have a life again.

(For children) it is so important to be able to participate in normal activities with their peers, at school and all the extra-curriculum activities that they have opportunities to be involved in.

### 3.2. Coping and Adapting to LT

#### 3.2.1. Resilience

HCP acknowledged the differing ways pediatric LT recipients experience the process of LT. HCP reflected on children's protective ability to adapt positively to a significant event such as life-saving LT. This ability, defined as resilience, was thought to be most notable in the younger aged patients and in those living with chronic liver disease leading to LT.

Like anything that happens in life, you can make the best of bad things that have happened in your life and learn from or you go through life being really negative. So this may even be predisposed by what the personality was beforehand.

For children that have received a transplant before the age of three, as far as they are concerned, they have always been transplanted.

#### 3.2.2. Acute Liver Failure

Pediatric acute liver failure as the indication for LT was viewed to hinder patients' opportunity to adjust adequately to such a life-changing event compared to other etiologies including chronic cholestatic liver diseases. HCP spoke about both the shorter duration, fulminant course of disease, and expedited waiting period prior to transplantation in the acute liver failure population as influencing posttransplant HRQoL and experience.

It depends on what their underlying reason for the liver transplant was. So for someone with fulminant failure who is totally fine, comes in devastatingly sick and has the transplant and doesn't even have time to think even about the transplant; to deal with all that is much different.

#### 3.2.3. The Surgical Scar

HCP commonly spoke about patients' difficulties dealing with their surgical scars, including doubts or decreased self-confidence to reveal their abdominal scars and fears of acceptability by others. Subsequently, pediatric LT recipients were perceived at risk for poor body image perception, low self-esteem, self-imposed social isolation, or avoidant behaviors. The adolescent female population was noted to be especially vulnerable to these struggles.

Teenage boys might like showing off their scars, their battle wounds (scars), as opposed to the girls who probably have a lot more body image issues.

Teenagers struggle with body image when they have to return to school with a scar (after LT). They don't want to go back to their swimming team or change into their bathing suits.

Some patients are really adept at keeping personal information away from their friends and they don't want them to know about it. It might be a quality of life issue for the child to work extra hard to make sure that your friends don't find out that you've had a transplant.

### 3.3. Living with a Transplanted Liver

#### 3.3.1. Clinical Care and Surveillance

Focus group participants highlighted the burden of clinical care and follow-up demands associated with post-LT care, including repeated blood work, clinic visits, liver biopsies, and imaging studies. HCP also highlighted the relevance of school absenteeism secondary to frequent clinic visits and hospitalization. School absenteeism, together with the possibility of impaired neurocognitive outcomes as sequelae of previous hepatic insufficiency and malnutrition, was thought to negatively influence school performance.

Children will have interruptions (of school attendance) depending on how frequently they have to return for tests or follow up visits.

Depending on how sick they were before transplant, patients are not accustomed to post-transplant care where you have to bring them in so often for the different tests.

I think they worry about being away from school again. I mean they have already missed a whole year of school and they are a year behind their peers.

#### 3.3.2. Medication-Related Problems

Issues regarding medication administration and medication associated adverse effects emerged as major barriers to treatment adherence and good HRQoL in LT recipients. Perceived barriers to medication administration included a lack of pediatric formulations, poor medication palatability, polypharmacy, frequency of dosing intervals, need for medication storage, or child's resistance to treatment. Cosmetic side-effects associated with the use of steroids, such as weight gain, hirsutism, or acne, were also thought to yield deleterious impacts on patients' body image and emotional functioning. HCP also reported on the long-term immunosuppression associated complications such as posttransplant lymphoproliferative disease, arterial hypertension, or renal dysfunction.

Some kids say that the number of medications that they are on is quite overwhelming.

For the teenagers, taking medications at specific times can be totally disruptive to their teenage lifestyle.

We always try to minimize medication side effects but depending on their post-transplant course that is not always possible and they develop kidney problems which causes them to have more medications.

#### 3.3.3. Restrictions and Overprotective Environment

HCP spoke about LT recipients being restricted in everyday activities such as physical activity, social gathering, or travelling. Such limitations were perceived to reflect either professional counseling or parental fears of infection secondary to immunosuppression therapy or concerns regarding traumatic graft injury. Additionally, healthy lifestyle counseling for LT recipients to avoid drugs and alcohol was thought to sometimes hinder patients to seeing themselves as equal to their peers or to engaging with peers relative to reasonable “at risk” activities associated with adolescent development. All of these were described as elements fostering an overprotective living context for LT recipients.

…families can become extremely overprotective, not allowing the child to be with other children because of concerns about infection.

Wanting to go out and really wanting to be like everybody else and having something that differentiates them from their peers is sometimes a lot for them.

They don't understand why they now have to be different. And different from their siblings too because siblings are allowed to do certain things and they aren't.

#### 3.3.4. Worries about the Future

HCP described patients' fears and anxieties regarding their future. Reported transplant recipients' concerns regarding their future health included fear of infection, rejection, and graft loss in addition to the fear of hospital readmission, procedural requirements, and intensified immunosuppression therapy. HCP also highlighted LT recipients' anxiety related to romantic partnering, having children, and fear of relocation to a distant place from their transplant center. Such fears and anxieties were ultimately perceived to have a negative impact on patients' mental health.

Some of the older children worry about rejection and the need to undergo a procedure and a receiving a course of steroids.

They worry about what being ill and hospitalized again. They also worry about what if their liver fails and even about mortality.

Teenagers and predominantly girls, will think not only about their own health but also about being in a relationship.

#### 3.3.5. Disease Self-Management

Poor disease self-management, dependency on their caregivers, and a lack of autonomy were perceived to be barriers to a smooth, incremental transition to adult care and jeopardizing follow-up and self-management in adulthood.

Children are fairly dependent on their parents for almost all of their care.

There is a group of patients that can't take control of their medical management and they get transferred to adult care and are just shell shocked that they actually have to go to the doctor's office on their own.

### 3.4. The Family Context

#### 3.4.1. Family as a Support System

In considering children's HRQoL after LT, HCP highlighted the family context in which children reside. HCP perceived the benefits of a robust family structure with parental cohesion able to support children throughout the continuum of the LT process and to positively impact patient outcomes. Professionals also described the profound impact of LT on patients' families, including negative consequences of LT on the above-mentioned family structure and cohesion, the risk of a parent losing her/his job due to the need for relocation to achieve proximity to tertiary care, or the need to provide family-based care.

I see a big support system between patients and their families, because they do not think that anybody else really gets what is going on or what they have been through.

Children may have the perception that having someone (mom) that does everything for them is a great quality of life.

Through the stresses of transplant the family falls apart and if we believe that the nuclear family is the best way to go through life, that has to have a negative impact on the quality of life.

I think transplant sometimes affects the families in the long term. For example, [patients] who have siblings and siblings have less attention because the mother is in the hospital with the sick child….

#### 3.4.2. Parental Stress and Family Coping

HCP underscored higher levels of stress and anxiety among parents of pediatric LT recipients. Focus group participants described the difficult emotions that parents experience during the LT listing process, transplant surgery, postoperative recovery period, long-term follow-up, and transition to adult care of their children. Parents' struggling to cope with their child's disease and treatment was perceived as a negative influence on their child's well-being.

How children react really comes down to how they have been brought up and how they have been parented. For example, parents can either create a positive out of having to get a procedure done or struggle with it. So the same procedure for a child can turn into two different experiences depending on the family environment.

In families that are very easy going and seem to adapt fairly well, often times the child has the same kind of coping skills and has a better quality of life.

Whatever the parents feel tends to be reflected in their child. For example, the families that are very anxious about things, that tends to be get transferred to the child and the child will become very anxious too.

## 4. Discussion

Qualitative studies on HRQoL after pediatric LT to date have focused on patient- and parent-reported perspectives. To the best of our knowledge, this is the first study providing HCP-reported perspectives on HRQoL in infants, children, and adolescents who have undergone LT. From the lens of HCP dedicated to the care of children with chronic liver disease and solid organ transplantation, attention should be placed on the promotion of patients' functioning, coping, and psychosocial well-being. The family context in which children undergo LT and the potential negative effects of parental stress on patients' HRQoL also emerged as key themes during focus group discussions.

Barriers to and facilitators of better HRQoL across all domains of HRQoL (physical health, mental health, social functioning, role functioning, and general health perceptions) were reported by HCP, in consistency with previous qualitative studies exploring patients' [12, 17, 18] and their parents' perspectives [17]. HCP perceived gains in physical health and a resumption of normal life activities as a vehicle to better outcomes of HRQoL following pediatric LT. Inevitable factors such as the need for long-term immunosuppression and the need for procedural and monitoring interventions were highlighted as key challenges for LT recipients. Additional concerns regarding patients' distress attributed to fears and anxieties around their future health were also identified, in alignment with recently reported high prevalence of mental health problems in young people after LT [18].

HCP dedicated to the care of pediatric LT patients participating in this study described the family burden related to caregiving demands, mobility impositions, social restrictions, and/or financial losses due to work absenteeism as key contributory variables to poor family functioning among families with a child who has undergone a LT. Family functioning, defined as the frequency of disruption of usual family activities, effectiveness of family communication and problem solving, and degree of family cohesiveness, has been investigated through quantitative research methods. These studies found no significant differences in family functioning between families of LT recipients and published norms for nonclinical families [19, 20]. However, pediatric solid organ recipients from healthier functioning family systems experience better quality of life [21]. Therefore, it remains important to characterize the phenomena of family functioning, to better identify those families who may benefit from targeted interventions.

The impact of LT affecting negatively parental psychosocial well-being and the interrelation between parental stress and children's well-being were discussed by HCP. These findings are in keeping with previously reported higher rates of depression, anxiety, and symptoms of posttraumatic stress disorder (PTSD) among parents of children either listed for LT and in parents of children who have undergone LT [22–24]. It is important to note that parental stress has been associated with impaired clinical outcomes after LT, including poor adherence to treatment and poor health outcomes [20]. Furthermore, findings of a recent study demonstrated significant associations between family strain in parents and emotional and behavioral disturbances in children with a LT, in keeping with HCP views on the relation between parental distress and increased coping difficulties in their children [25]. While it is intuitive to think that high level of distress in parents may lead to greater adaptation struggle in their children relative to adjustment to illness, greater understanding of how this emerges and what mediates outcomes is needed in further study.

### 4.1. Study Limitations

Although the sample size was small (*n* = 13), this study enabled issues important to HRQoL following pediatric liver transplantation to be examined in detail and in depth and enabled subtleties and complexities to be discussed by a wide spectrum of interdisciplinary highly experienced HCP delivering specialized pediatric LT care. Our sample only included HCP from two Canadian health care tertiary institutions, which may limit the transferability of findings to other health care organizations. Nevertheless, the themes identified from focus group discussions are likely to be relevant to other pediatric LT recipients. This study is also limited by the lack of male HCP participants, which may be reflective of gender ratio at the study institutions. Therefore, this study is not claiming to be reflective of the more general pediatric HCP population. Limitations of focus group studies include heir susceptibility to bias, because group and individual opinions can be swayed by dominant participants or by the moderator. In this study, this limitation was overcome with the participation of a trained and skilled moderator in each group.

### 4.2. Implications for Clinical Practice

With large pediatric LT programs now achieving excellent patient and graft survival outcomes, it becomes increasingly important to ensure that optimal subjective outcomes are also achieved. In achieving such, early interventions aimed at promoting adaptive coping strategies and fostering patients' resilience should be implemented. Moreover, it is also recommended that HCP will remain dedicated to promoting patient mental health and psychosocial well-being [26]. The recently validated disease-specific HRQoL tool (PeLTQL) exemplifies one of the clinically available and nonstigmatizing tools that HCP can use to screen for patient well-being and mental health concerns including anxiety and childhood-depression [27].

Based on the findings of this study, a shift in the orientation of pediatric LT care from a patient-centered to a collaborative family-centered care model is pivotal. In delivering family-centered care, HCP responsibilities would include screening, assessment, and referral of parents with physical, emotional, or social challenges that might negatively affect the health and emotional or social well-being of the transplant candidate or LT recipient.

## 5. Conclusion

The critical insights from professionals obtained in this study play a complementary role in generating understanding of the elements contributing to HRQoL after LT and the framework in which HRQoL is being measured. HCP reported on patients' adaptive coping skills and their family context as key areas for intervention. These findings may allow us to improve our current model of care, through delivering proactive psychosocial support, which consists of attention to processes of resilience, the promotion of mental health, and family-centered care.

## Figures and Tables

**Box 1:**
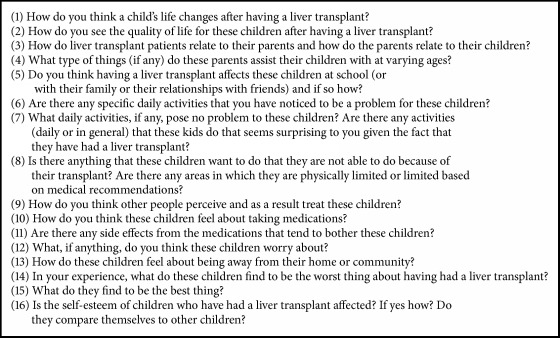
Semistructured focus group questions.

**Table 1:** Characteristics of focus group participants.

*Participants recruited (n)*	13
*Female sex (n)*	13
*Age range (n)*	
25–35 years	6
36–45 years	6
46 years and older	1
*Years of expertise in LT (n)*	
Less than 5 years	1
5 years or more	12
*Professional category (n)*	
Transplant surgeon	1
Pediatric hepatologist	2
Nurse practitioner	1
Registered nurse	3
Transplant coordinator	1
Pharmacist	1
Physiotherapist	2
Occupational therapist	1
Social worker	1

## Abbreviations

HCP: Health care providers
HRQoL: Health-related quality of life
LT: Liver transplantation.
